# False-positive Xpert^®^ Xpress SARS-CoV-2 assay in an emergency room and trauma center: A retrospective chart review study

**DOI:** 10.15537/smj.2022.43.8.20220317

**Published:** 2022-08

**Authors:** Hyerim Kim, Soeun Jeon, Sun Hack Lee, Hyun-Su Ri, Hyeon-Jeong Lee, Jeong-Min Hong, Sung In Paek

**Affiliations:** *From the Department of Laboratory Medicine (Kim); from Biomedical Research Institute (Kim, Jeon, S. H. Lee, H-J. Lee, Hong); from the Department of Anesthesia and Pain Medicine (Jeon, H-J. Lee, Hong, Paek); from the Department of Internal Medicine (S. H. Lee), Division of Cardiology, Pusan National University Hospital, Pusan National University School of Medicine, Busan, and from the Department of Anesthesia and Pain Medicine (Ri), Kyungpook National University, School of Medicine, Daegu, Korea.*

**Keywords:** COVID-19, SARS-CoV-2, COVID-19 nucleic acid testing, polymerase chain reaction, false positive reactions

## Abstract

**Objectives:**

To review reports false-positive Xpert results in an emergency room and trauma center.

**Methods:**

Patients’ data with false-positive Xpert results from November 2020 to February 2022 at Pusan National University Hospital, Busan, Republic of Korea, were extracted from the electronic medical records.

**Results:**

The positive predictive value of Xpert was 40%. Of the 12 patients with false-positive results, 5 (41.7%) were re-positives (such as, patients recovered from coronavirus disease-19 [COVID-19]), and 4 (33.3%) had head or facial trauma. Two out of 4 head or facial trauma cases had documented sample contamination with blood.

**Conclusion:**

We found a high incidence of false-positive Xpert results among patients who recovered from COVID-19 and those with head or facial injury. Careful history taking for COVID-19 and physical examination of the sample collection site is essential before Xpert analysis.

**R**eal-time reverse-transcription polymerase chain reaction (rRT-PCR) based on viral ribonucleic acid amplification technique is the gold standard for the detection of severe acute respiratory syndrome coronavirus-2 (SARS-CoV-2).^1^ However, as an on-site point-of-care testing, conventional rRT-PCR is time-consuming, owing to its amplification time.^1^

Xpert^®^ Xpress SARS-CoV-2 assay (Xpert; Cepheid, Sunnyvale, CA, USA) is an automated, cartilage-based, rapid rRT-PCR designed to deliver sample-to-result within an hour.^1,^2^^ This state-of-the-art diagnostic tool has been predominantly applied in acute care settings to support rapid decision making in patients with life-threatening, time-limited, and urgent/emergent care needs.1 However, Xpert is not completely validated and could be inaccurate, particularly in bloody and viscous specimens.^3^

False-positive coronavirus disease-19 (COVID-19) test results, although issued less frequently than false-negatives, could result in significant adverse outcomes, including additional investigations, unnecessary consumption of material and labor resources, potential exposure risk of non-infected patients to the COVID-19 cohort area, and loss of precious time for medical or surgical interventions, especially in emergency room and trauma center settings.^4^ In this retrospective chart review study, we report false-positive Xpert results in an emergency room and trauma center. We also carried out a relevant literature review.

## Methods

The Institutional Review Boards of Pusan National University Hospital, Busan, Korea, approved and exempted this study from the requirement of informed consent (ID: 2203-014-113). This study complies with the principles of the Helsinki Declaration. The study subjects were patients with false-positive Xpert results, obtained from November 2020 to February 2022 at Pusan National University Hospital, Busan, Korea.

A patient with a false-positive Xpert result was defined as a patient with a positive Xpert result and a negative result in a subsequent confirmatory rRT-PCR test and determined not to require COVID-19 isolation by an infectious disease specialist. Xpert was carried out for patients who met the following 2 inclusion criteria: i) visited the emergency room or trauma center; and ii) in life-threatening condition or requiring surgical intervention that could not be delayed for more than 6 hours. As a confirmatory test, rRT-PCR testing was carried out for patients who had positive Xpert results. Xpert was carried out using nasopharyngeal swabs, and confirmatory rRT-PCR testing was carried out using both nasopharyngeal and oropharyngeal swabs. All specimens were collected by qualified health care providers.

The following data were retrieved from the electronic medical records: i) demographics and disease characteristics (American Society of Anesthesiologists physical classification, age, gender, height, weight, chief complaints, clinical manifestations, and comorbidities); ii) vital signs, laboratory and image findings during presentation; iii) presence of physical injury and trauma; iv) history of COVID-19; v) total isolation period for COVID-19 in our hospital; vi) specimen contamination with blood (whether sample contamination with blood was documented in the clinical laboratory footnote for patients with physical injury or trauma); VII) final diagnosis and clinical outcomes; and VIII) surgical delays (total delay in surgical intervention due to false-positive results in Xpert). The following medical records were excluded: patients with negative, invalid, or true positive results.

All analytical procedures were carried out by qualified clinical laboratory technologists. Emergency screening test for SARS-CoV-2 (rapid rRT-PCR) was carried out with Xpert and GeneXpert. The USA Food and Drug Administration (FDA) approved a rapid molecular diagnostic test for Cepheid, which can diagnose COVID-19 infection in 45 minutes.^2^ Xpert intended for the qualitative detection of the nucleocapsid gene (especially, primer to N2 region) and envelope genes of SARS-CoV-2 using GeneXpert system.^2^

For patients with positive Xpert test results, a confirmatory nucleic acid amplification test (NAAT) was carried out with PowerChekTM 2019-nCoV Real-time PCR (KogeneBiotech, Seoul, Korea) based on rRT-PCR (that has emergency use authorization from the USA FDA). The PowerChekTM 2019-nCoV Real-time PCR, a single-tube multiplex rRT-PCR assay, can simultaneously detect the open reading frame 1ab and envelope genes of SARS-CoV-2 under the 7500 Real-Time PCR system (Applied Biosystems, Waltham, MA, USA).^5^ The test result was considered positive when all target genes were detected together using variable specimens (sputum, bronchoalveolar lavage, bronchial washing, nasopharyngeal aspirate, combined nasopharyngeal/oropharyngeal swab, and endotracheal aspirate). The results were interpreted as positive when an exponential fluorescence curve crossed the threshold line at or before 38 cycles (cycle threshold ≤38) in 2 hours.^5^

### Statistical analysis

MedCalc software, version 18.11.6 (MedCalc Software bvba, Ostend, Belgium) was used for statistical analysis. The variables are reported as absolute numbers (percentages) or ranges.

## Results

**Figure 1:**
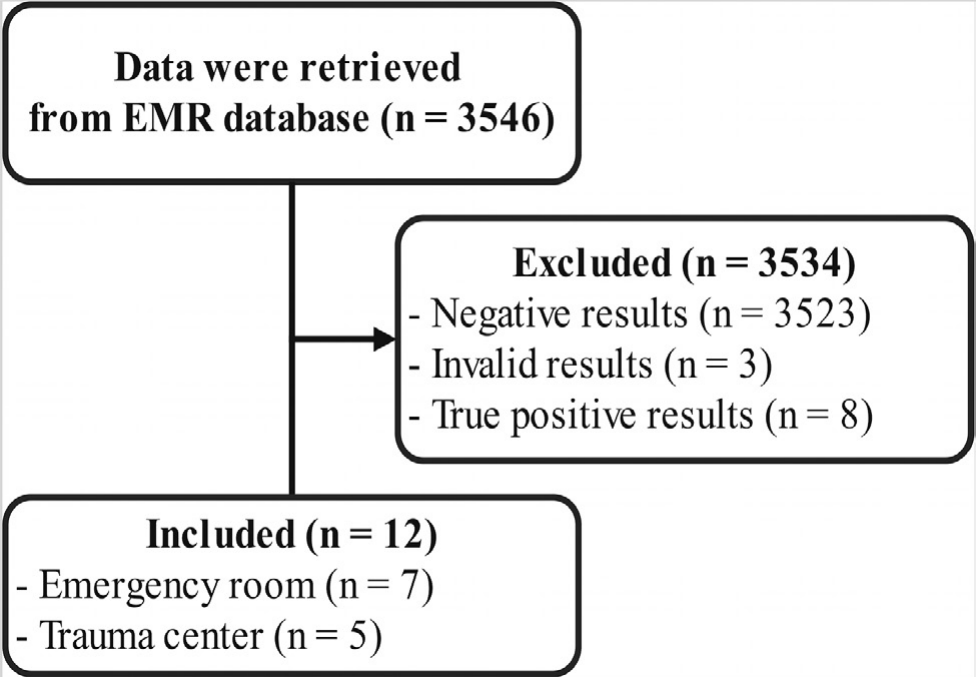
- Study flow chart. EMR: electronic medical record.

Of the 3546 patient records extracted, 3534 were excluded: negative Xpert results (n=3523), invalid Xpert results (n=3), and true-positive results (positive results for both rapid and confirmatory rRT-PCR tests; n=8; Figure 1). All patients with invalid Xpert results were retested using confirmatory rRT-PCR, and all had negative results.

The cases are summarized in Tables 1 & 2. Of the 12 included patients, 7 patients visited the emergency room and 5 visited the trauma center. Of the emergency room cases, 5 were re-positive and they had recovered and were discharged from COVID-19 isolation before admission (41.7% of the total and 71.4% of the emergency room patients). Of the trauma center patients, 4 had head or facial trauma (33.3% of the total and 80% of the trauma center patients); 2 cases of sample contamination with blood were documented in the clinical laboratory footnotes (16.7% of the total and 40% of the trauma center patients), and the remaining cases were not clearly documented. Only 3 (25%) patients had pulmonary manifestations. Five patients required surgical intervention, of which 3 had delays in surgical intervention due to false-positive Xpert results (range: 4-5.5 hours). The mean isolation period due to false-positive Xpert results was 12 hours (range: 3-24 hours).

**Table 1:** Patients characteristics, laboratory findings, vital signs, and chest x-ray findings at the time of the presentation.

Case No.	Patients characteristics					Laboratory findings					Vital signs			CXR findings
	ASA	Age (yr)	Gender	Ht (cm)	Wt (kg)	Hb (g/dL)	Plt (10^3^/µL)	WBC (10^3^/µL)	Lymphocyte (%)	CRP (mg/dL)	SpO_2_ (%)	SBP (mmHg)	BT (°C)	
1	I	20	F	168	53	13.6	190.0	3.8	32.9	1.59	100	100	36.6	NALL
2	III	75	F	160	55	12.8	315.0	20.0	8.0	0.06	98	180	35.7	NALL
3	II	50	F	169	62	13.7	161.0	3.5	9.8	0.13	100	150	37.6	Tiny calcified nodule in RUL
4	II	59	M	166	71	14.4	395.0	19.1	8.4	0.13	100	140	36.8	Rt. 6-9^th^ rib fractures, pulmonary congestion
5	II	48	F	155	55	12.1	139.0	19.6	17.1	0.04	95	60	36.0	Lt. 1-7^th^ rib fractures and hemopneumothorax
6	IV	70	F	160	59	12.1	231	9.9	11.8	0.05	90	200	36.7	Cardiomegaly, Pulmonary congestion and edema in both lung fields
7	II	32	F	163	63	11.7	258	5.8	8.0	-	98	110	37.2	NALL
8	II	22	F	168	66	13.5	392	11.1	12.6	0.16	100	100	37.6	NALL
9	IV	71	M	180	72	13.8	97	8.5	41.3	0.05	99	160	36.4	R/O both lung contusion
10	IV	82	F	150	45	10.1	326	13.0	6	1.24	50	110	36.0	R/O aspiration pneumonia
11	IV	16	F	160	50	8.5	215	8.9	77.1	0.37	Undetected		-	Subtle haziness in Rt. lung
12	II	26	M	180	80	15.2	350	11.8	21.4	0.02	99	100	36.6	NALL

Reference ranges for laboratory tests: Hb: 13.5-17.5 g/dL, Plt: 140-420 103/µL, WBC: 3.8-11.0 103/µL, lymphocyte: 20-48.0%, and CRP: 0-0.5 mg/dL. ASA: American Society of Anesthesiologists physical classification, F: female, M: male, Hb: hemoglobin, Plt: platelets, WBC: white blood cells, CRP: c-reactive protein, SpO_2_: percutaneous arterial oxygen saturation, SBP: systolic blood pressure, BT: body temperature, CXR: chest x-ray, NALL: no active lung lesion, RUL: right upper lobe, R/O: rule out, (-): not measured, No.: number

**Table 2:** - Clinical characteristics, comorbidities, specimen contamination, history of coronavirus disease-19, and clinical courses.

Case No.	Department	Chief complaint	Comorbidities	Head/facial trauma	Specimen contamination by blood*	COVID-19 history	Pulmonary symptoms	Isolation period**
1	ER	Low abdominal pain	None	None	-	None	None	4 hours
	Final diagnosis: pelvic inflammatory disease. Outcomes: outpatient follow-up without hospitalization.							
2	ER	Decreased mentality (semicoma)	Hypertension Cerebral aneurysm	None	-	None	None	5.5 hours
	Final diagnosis: Lt. middle cerebral artery aneurysm rupture. Outcomes: after confirming the negative COVID-19 result of the real-time RT-PCR test, aneurysm coiling and aneurysm clipping with decompressive craniectomy were carried out; expired on POD11 (surgical delays***: 4 hours).							
3	ER	Chest pain, fever	Total thyroidectomy state (due to thyroid cancer; TFT: n-s)	None	-	Recovered COVID-19 patient (isolation lifted 6 days ago)	Mild dyspnea	4 hours
	Final diagnosis: R/O chostochondritis. Outcomes: outpatient follow-up without hospitalization.							
4	Trauma center	TA (bicyclists),chest pain	None	Facial abrasion	Not reported	None	Mild dyspnea	13 hours
	Final diagnosis: facial abrasion; Rt. 6-9th rib fx.; Rt. minimal pneumothorax. Outcomes: after admission, conservative treatment was carried out; discharged after 3 days							
5	Trauma center	Crushing injury	None	Panfacial fx.	Bloody sample	None	Dyspnea	5 hours
	Final diagnosis: panfacial fracture; Lt. upper arm amputation; Lt. 1-7th rib fx.; Lt. scapular fracture. Outcomes: after confirming the negative COVID-19 result of the confirmatory rRT-PCR test, Lt. arm wound closure was carried out; open reduction and internal fixation were carried out for pan facial fracture, scapular fracture, and rib fracture; discharged on POD26 (surgical delays: 5.5 hours).							
6	ER	Dyspnea	Uncontrolled hypertension, ESRD on HD, A-fib, and CHF	None	-	Recovered COVID-19 patient (isolation lifted 14 days ago)	Dyspnea	one day
	Final diagnosis: CHF exacerbation (EF 50%->20%) and pulmonary edema. Outcomes: after emergency hemodialysis, cardiopulmonary function recovered; discharged after 4 days							
7	ER	Preterm labor	Intrauterine pregnancy (30weeks)	None	-	Recovered COVID-19 patient (isolation lifted 8 days ago)	None	one day
	Final diagnosis: preturm labor. Outcomes: emergency vaginal delivery was carried out in the ER; discharged after 2 days							
8	ER	Decreased mentality (drowsy)	Mental retardation, bipolar disorder	None	-	Recovered COVID-19 patient (isolation lifted 45 days ago)	None	one day
	Final diagnosis: catatonia. Outcomes: hospitalization and medication change, discharged after 29 days							
9	Trauma center	TA (pedestrian), decreased mentality (stupor)	DM	Skull fx.	Not reported	None	None	3 hours
	Final diagnosis: traumatic SAH and SDH; multiple skull fx. Outcomes: after confirming positive rapid rRT-PCR test results, craniectomy was carried out in the negative pressure operating room with protective equipment (PAPRs), expired on POD3 (surgical delays: none).							
10	ER	Desaturation (SpO_2_ 50%, room air)	Tracheostomy state for management of COVID-19 ARDS (33 days ago)	None	-	Recovered COVID-19 patient (isolation lifted 9 days ago)	Desaturation	13 hours
	Final diagnosis: tracheostomy associated pneumonia. Outcomes: removes secretions by suctioning the tracheostomy tube. After desaturation improved, the patient was transferred to a community hospital.							
11	Trauma center	TA (motorcyclist), cardiac arrest.	None	Skull and panfacial fx.	Bloody sample	None	None	20 hours
	Final diagnosis: traumatic SAH and SDH; skull fx.; panfacial fx.; Rt. pneumothorax; Rt. femur fx. Outcomes: after one cycle of CPR, the patient had a return of spontaneous circulation. Without confirming the rapid rRT-PCR test results, decompressive craniectomy was carried out in a negative pressure operating room after wearing PAPRs; expired on POD1 (surgical delays: none).							
12	Trauma center	Drunken state, glass laceration injuries on both arm and abdomen.	None	None	Not reported	None	None	5 hours
	Final diagnosis: glass laceration injuries on both upper arm and abdomen. Outcomes: after confirming the negative COVID-19 result of the confirmatory rRT-PCR test, wound closure was carried out; discharged on POD7 (surgical delays: 5 hours).							

* Whether sample contamination by blood was documented in the clinical laboratory footnote for patients with physical injury or trauma.

** Total isolation period for COVID-19 in our hospital.

*** Total delay in surgical intervention due to false-positive results in rapid rRT-PCR for COVID-19. ER: emergency room, POD: postoperative day, TFT: thyroid function test, n-s: nonspecific findings, R/O: rule out, TA: traffic accident, fx: fracture, ESRD: end-stage renal disease, HD: hemodialysis, CHF: chronic heart failure, EF; ejection fraction, DM; diabetes mellitus, rRT-PCR: real-time reverse-transcription polymerase chain reaction, SAH: subarachnoid hemorrhage, SDH: subdural hemorrhage, PAPR: powered air purifying respirator, ARDS: acute respiratory distress syndrome, SpO_2_: percutaneous arterial oxygen saturation

## Discussion

The COVID-19 pandemic has increased the demand for point-of-care diagnostic tools to improve patient throughput and to support timely decision-making.^6^ Conventional rRT-PCR is considered the current standard diagnostic test for detecting SARS-CoV-2; however, it requires several hours and skilled human resources.^2^ Accordingly, Xpert, a type of rapid rRT-PCR test, has been designed to reduce the time required for conventional rRT-PCR testing and deliver sample-to-result within an hour. Xpert is an automated cartilage-based diagnostic tool that streamlines specimen processing, nucleic acid extraction, amplification, and amplicon detection by integrating these processes into a single cartridge operation.^7^

While there are some controversies regarding the validity of rapid rRT-PCR tests, a meta-analysis reported that the sensitivity of rapid rRT-PCR test was 95.1% (95% confidence interval [CI]: [90.5-97.6]) and specificity of rapid rRT-PCR test was 98.8% (95% CI: [98.3-99.2]).^3,8^ In a subgroup analysis, the sensitivity of Xpert was 100% (95% CI: [88.1-100]) and specificity of Xpert was 97.2% (95% CI: [89.4-99.3]); the estimated positive predictive value (PPV) was 65% at 5% prevalence of COVID-19, estimated PPV was 80% at 10% prevalence of COVID-19, and estimated PPV was 90% at 20% prevalence of COVID-19. ^8^

However, in our retrospective chart review study, the PPV of Xpert was only 40% (8 true-positive and 12 false-positive results). Considering that the prevalence of COVID-19 in South Korea was approximately 5.2% as of February 2022, our results considerably deviate from previous findings.^8,9^ This could be because our study was based on data from patients visiting emergency and trauma rooms, not the general population. Therefore, our study results should be limited to the emergency department and trauma department settings, and further studies should be carried out on the general population. Additionally, considering 29% of rapid antigen test results are false-positive, the higher false-positive rate of rapid rRT-PCR in our study could be due to re-positive cases after discharge from COVID-19 isolation and specimen contamination by blood in patients with head or facial injury.^10^

Of the 12 patients with false-positive results in our study, 5 recovered and were discharged from COVID-19 isolation before admission (such as, re-positive rRT-PCR for SARS-CoV-2). The Korean Centers for Disease Control and Prevention (KCDC) carried out a large-scale investigation on recovered COVID-19 patients (n=285) and reported that the rate of re-positive rRT-PCR test for SARS-CoV-2 in recovered COVID-19 patients was 25.9-48.9%, while the average time from discharge to re-positive result was 14.3 days (range: 1-37 days).^11^ They also carried out viral cell culture (n=108), and no case of virus isolation was observed.^11^ Xing et al^12^ reported serial fluctuating rRT-PCR test results in recovered COVID-19 patients, which resulted in confusion. Re-positive results after COVID-19 recovery can stem from inherent limitations of the nucleic acid amplification technology, including both rapid and confirmatory rRT-PCR tests.^13^ While rRT-PCR testing detects the presence of viral gene segments, it does not clarify whether the virus is intact or infective.^13^ Human respiratory epithelial cells have a half-life of up to 3 months; the remnants of SARS-CoV-2 genetic material in these epithelial cells can be identified using rRT-PCR testing even 1-2 months after full recovery from COVID-19.^13^ Based on this evidence, the KCDC recently concluded that re-positive SARS-CoV-2 results are not infectious or reactivated in case of i) re-positive result within 45 days of initial diagnosis; ii) no exposure history; iii) no clinical manifestation.^14^ These recently revised guidelines suggest that scrutiny of COVID-19 history should precede screening tests.^14^

Of the 12 patients with false-positive results observed in our study, 4 had head or facial injury. Only nasopharyngeal swabs were used for Xpert following the manufacturer’s instructions; thus, specimens from patients with head or facial injuries could be contaminated with blood.^7^ We searched the relevant EMR database and found that 2 of 4 samples of patients with head or face injuries had documented contamination with blood in the clinical laboratory footnotes; in the other 2 cases, sample contamination by blood was not clearly documented because it was not mandatory to record the status of nasopharyngeal swabs in our clinical laboratory. Contamination of the specimen by blood causes inaccuracy in various SARS-CoV-2 diagnostic tests. Mouliou et al^3^ reported that bloody and viscous specimens could yield misleading rapid rRT-PCR test results. Considering rapid antigen tests based on lateral flow technology, Kahn et al^15^ reported that blood-contaminated samples could cause false-positive results and estimated that 32.2% of these false-positives were blood-contaminated samples. According to the manufacturer’s instructions, Xpert is only validated with nasopharyngeal swab specimens.^7^ Therefore, the performance of Xpert with other specimen types should be evaluated to use this assay as a point-of-care diagnostic test in patients with head or facial injuries.

### Study limitations

First, we used EMR data before the Omicron shift in South Korea. Therefore, further evaluation is needed to reflect the change in the prevalence of COVID-19 in South Korea after the omicron-dominant wave. Second, our study had limited population of emergency and trauma department. Third, we carried out a retrospective chart review; thus, our findings do not provide definite conclusions regarding the cause-effect relationship between potential contributing factors (COVID-19 history and blood-contamination) and outcomes (false-positive result in Xpert). However, our study highlights the potential contributing factors for diagnostic errors in real-world clinical settings (beyond well-controlled laboratory-based research).

In conclusion, we found a high incidence of false-positive Xpert results in patients who recovered from COVID-19 and those with head or facial injury. Careful history taking for COVID-19 and physical examination of the sample collection site are essential before Xpert analysis. Further well-designed studies should be carried out to validate the performance of Xpert using non-nasopharyngeal specimens to apply Xpert as a point-of-care diagnostic test in patients with head or facial trauma.
